# Spin-Independent Plasmonic Lens

**DOI:** 10.1186/s11671-019-2990-2

**Published:** 2019-05-07

**Authors:** Guoqun Li, Yuqing Sun, Sen Wang

**Affiliations:** Shandong Provincial Engineering and Technical Center of Light Manipulations & Shandong Provincial Key Laboratory of Optics and Photonic Device, College of Physics and Electronics, Shandong Normal University, Jinan, 250014 China

**Keywords:** Surface plasmons, Spiral phase, Spin, Focusing, Transverse shift

## Abstract

For the semicircular plasmonic lens, the spiral phase is the origin of the spin-dependent surface plasmon polariton (SPP) focusing. By counterbalancing the spin-dependent spiral phase with another spiral phase or Pancharatnam-Berry phase, we realized the SPP focusing independent from the spin states of the excitation light. Analyses based on both Huygens-Fresnel principle for SPPs and numerical simulations prove that the position, intensity, and profile of the SPP focuses are exactly the same for different spin states. Moreover, the spin-independent SPP focusing is immune from the change of the radius, the central angle, and the shape of the semicircular slit. This study not only further reveals the mechanism of spin-dependent SPP devices but also provides effective approaches to overcome the influence of spin states on the SPPs field.

## Introduction

In the three-dimensional (3D) free space, optical lenses play an indispensable role in molding the flow of light, such as focusing, imaging, and optical Fourier transform (FT). However, the inherent limitations of conventional lenses are also gradually unveiled. Due to the diffraction of light, the transversal full width at half maximum of a focus is no less than about half a wavelength *λ*/(2*n* sin *α*), which hinders the realization of superresolution lithography and microscopy [1–3]. As for the optical FT relation between the front and back focal planes, the speed of the transformation is restricted by the thickness and focal length of the lens [4]. Above all, compared with the wavelength of light, the volume of the lens is bulky because of the curved surface used to achieve gradual phase accumulation [5–7]. And that is incompatible with the increasing demand for miniature and integrated optical devices in research and applications [8–10].

Surface plasmon polaritons (SPPs) which are hybrid modes of phonons and electronic oscillations propagating along the two dimensional (2D) metal/dielectric interface can be an effective tool to overcome the above limitations [11–17]. With the subwavelength feature, SPPs can be easily focused to a subwavelength spot [18–21]. As the counterpart of the optical lens in the 3D space, semicircular slit plasmonic lens cannot only focus SPP fields but also perform SPP FT with a much faster speed in a 2D plane [4]. Besides, in order to effectively excite SPPs, the width of the slit is smaller than the wavelength of incident light. Nevertheless, the focusing of SPPs generated by the semicircular slit strongly depends on the spin states of the incident light [22–25]. For left circularly polarized (LCP) and right circularly polarized (RCP) incident light, the focal spots of SPPs will experience spin-dependent transverse shifts, which is distinctive from the focusing of circularly polarized light in the free space. Since the study of the spin-dependent semicircular SPPs lens in 2008 by Hasman et al. [22–24], various mechanisms have been proposed to accomplish the spin-dependent SPP focusing [26–28]. The basic principle relies on the spin-dependent phase distribution accomplished by steering the orientation angles of subwavelength slits. Moreover, spin-dependent SPP excitation [29], SPP vortex [30], SPP hologram [31], SPP Bessel beam [32], and SPP Airy beam [33] have been demonstrated. Overall, spin-dependent SPP devices have been extensively studied. It is obvious and normal that the spin states of excitation light can influence the functionality of SPP devices because even the SPPs excited by a single subwavelength slit or hole depend on the spin states [24, 26, 28, 33]. However, on the contrary, is it possible to avoid the influence of spin states on the SPPs field and make the SPPs lens spin-independent?

The SPPs generated by a semicircular slit are imprinted with a spin-dependent spiral phase exp(*iσ*_±_*θ*), where the spin states *σ*_±_ =  ± 1 represent LCP and RCP light, respectively [22–25]. In this paper, we propose a global approach and a local approach to eliminate the influence of the spiral phase and achieve spin-independent SPPs focusing. The global approach deals with the semicircular slit wholly and cancels out the spiral phase by adding an opposite semicircular slit which can introduce an inversed spiral phase. Regarding the semicircular slit as the constitution of subwavelength slits, the spiral phase can be counterbalanced locally with Pancharatnam-Berry phase which is tuned by changing the orientation angle of the slit. The spin-independent SPP focusing is analyzed and verified with the Huygens-Fresnel principle for SPPs as well as numerical simulations. The robustness of the proposed approaches is tested by changing the radius, central angle, and shape of the semicircular slit. Compared with previous spin-dependent SPP devices [26–33], the focusing of SPPs here is independent from the spin states of the excitation light, which could improve the stability of the SPP lens.

## Results and Discussions

### Spin-Independent Plasmonic Lens Consisted of Double Semicircular Slits

**Fig. 1:**
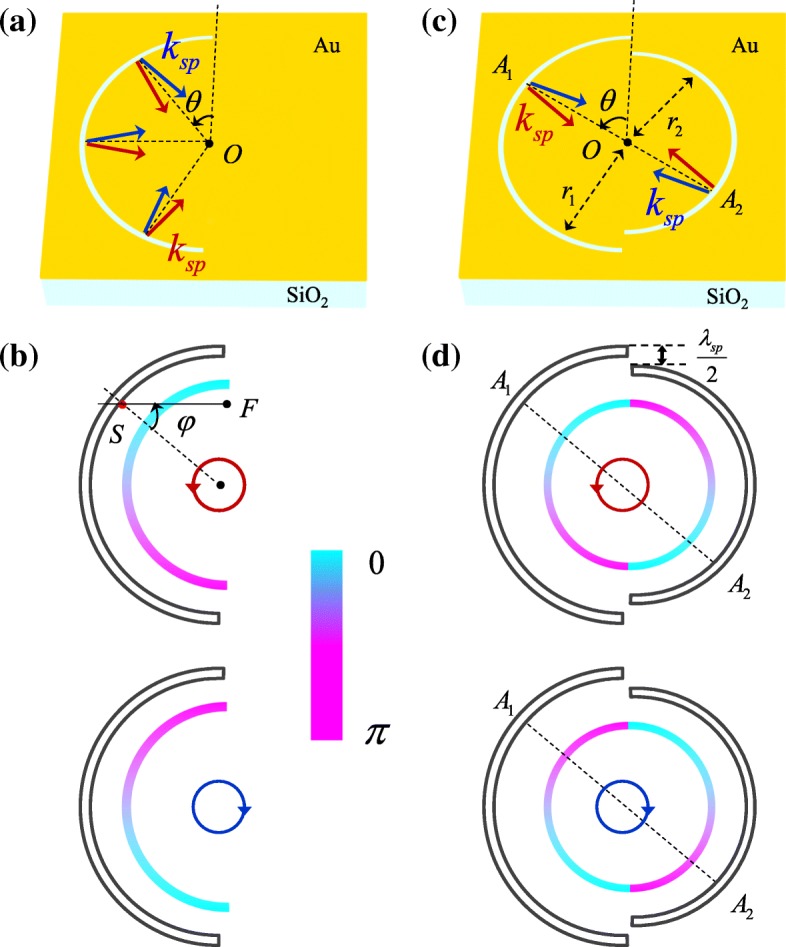
Schematic diagram of the semicircular slit plasmonic lens (**a**) and the spin-independent SPP lens consisted of two semicircular slits (**c**). With the illumination of LCP and RCP light, the excited SPPs will experience spin-dependent spiral phases (**b**). Adding another semicircular slit can introduce an extra spiral phase, and the two spiral phases can cancel out each other when *r*_1_ − *r*_2_ = *λ*_sp_/2 (**d**)

For semicircular slit plasmonic lens illuminated by left circularly polarized (LCP) and right circularly polarized (RCP) incident light, the spiral phases increase from 0 to *π* counterclockwise and clockwise, respectively, as schematically shown in Fig. 1b. The spiral phase results from the interaction between the circularly polarized light and anisotropic nanoscale structure [23]. Circularly polarized light is the synthesis of the horizontally polarized and vertically polarized light with a *π*/2 phase difference. The SPPs excited by the two linear components can be expressed as sin*θ* and cos*θ*, respectively [25]. Thus, the SPP field generated by circularly polarized light is sin*θ* + exp(*iσ*_±_*π*/2) cos *θ* = exp(*iσ*_±_*θ*). Without the spiral phase, the wavefront of SPPs would be parallel to the semicircular slit and the SPP wavevector *k*_sp_ would be along the radial direction. However, the spiral phase corresponds to a spiral wavefront and the SPP wavevector will deviate from the radial direction, illustrated by the red and blue arrows in Fig. 1a. And, ultimately, the spiral phase results in the transverse shift of the SPP focus [22, 23, 25]. It is obvious that the spin-dependent spiral phase, which is the origin of the spin controlled SPP focusing, needs to be eliminated to realize the spin-independent SPP lens.

Adding another semicircular slit to introduce additional spiral phase could be a solution. When the two semicircular slits are on the same side, the two spiral phases cannot cancel each other out. Thus, the semicircular slit should be added on the opposite side. Figure 1c schematically shows the structure of the SPP lens consisted of two semicircular slits with different radius *r*_1_ and *r*_2_. The excited SPP fields along the left and right semicircular slits can be correspondingly expressed as:

1 $$ {E}_{\mathrm{sp}}^{\mathrm{L}}\left({r}_1,\theta \right)=\exp \left(i{\sigma}_{\pm}\theta \right),\left(0\le \theta \le \pi \right), $$

2 $$ {E}_{\mathrm{sp}}^{\mathrm{R}}\left({r}_2,\theta \right)=\exp \left(i{\sigma}_{\pm}\theta \right),\left(\pi \le \theta \le 2\pi \right). $$

There exists a *π* phase difference between the spiral phases generated by two semicircular slits. Particularly, when the radiuses satisfy Δ*r* = *r*_1_ − *r*_2_ = *λ*_*sp*_/2, *k*_sp_Δ*r* = *π* could just compensate the *π* phase difference between the two spiral phases. As presented in Fig. 1d, the corresponding phase of SPPs is central symmetry. Concretely, the phase of SPPs generated from the point *A*_1_ is the same as the phase of SPPs generated from the symmetrical point *A*_2_. And the SPPs generated by *A*_1_ and *A*_2_ will interfere constructively in the center, so do the other points along the semicircular slits. Accordingly, the SPPs generated by the two semicircular slits will be focused in the center without transverse shift. When the spin states of the incident light are changed, the left and right spiral phases will be reversed simultaneously and remain to be central symmetry. Therefore, the SPPs excited by both LCP and RCP light can be focused in the center of the semicircular, which indicates the spin-independent feature of the plasmonic lens.

**Fig. 2:**
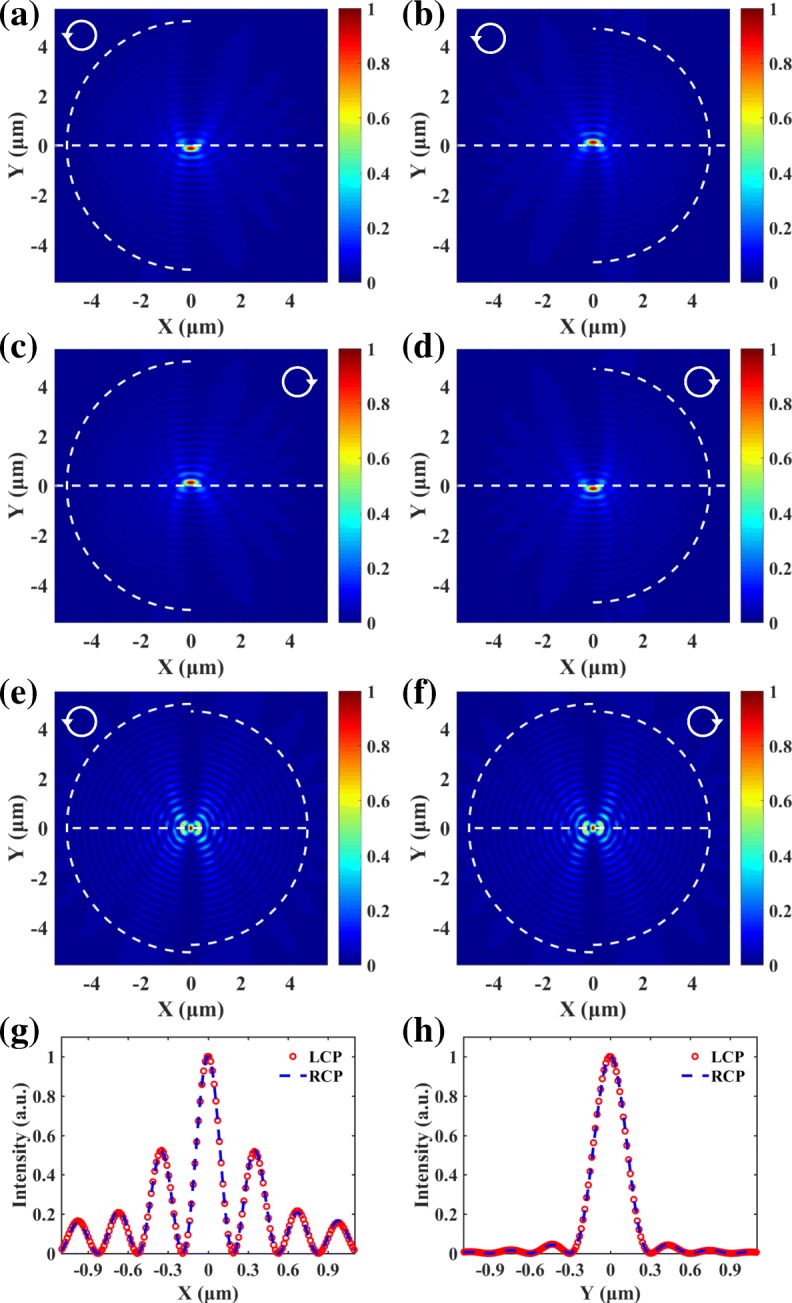
For LCP light, the SPP focuses generated by the left semicircle slit (**a**) and the right semicircle slit (**b**) shift downward and upward, respectively. For RCP light **c** and **d**, the positions of the SPP focuses are reversed. **e**, **f** The SPP focuses generated by spin-independent plasmonic lens are all in center for LCP and RCP light. **g**, **h** The transversal and longitudinal distributions of the SPP focuses

The performance of the spin-independent plasmonic lens is analytically examined with the Huygens-Fresnel principle for SPPs [34, 35]. In the polar coordinate system, the SPP fields generated by the left and right semicircular slits can be respectively expressed as:

3 $$ {E}_{\mathrm{sp}}^{\mathrm{L}}\left(\rho, \theta \right)=-\frac{i}{\sqrt{\lambda_{\mathrm{sp}}}}{\int}_0^{\pi}\cos \varphi {E}_{\mathrm{sp}}^{\mathrm{L}}\left({r}_1,\theta \right)\frac{\exp \left({ik}_{\mathrm{sp}}d\right)}{\sqrt{d}}\exp \left( i\pi /4\right){r}_1 d\theta, $$

4 $$ {E}_{\mathrm{sp}}^{\mathrm{R}}\left(\rho, \theta \right)=-\frac{i}{\sqrt{\lambda_{\mathrm{sp}}}}{\int}_{\pi}^{2\pi}\cos \varphi {E}_{\mathrm{sp}}^{\mathrm{R}}\left({r}_2,\theta \right)\frac{\exp \left({ik}_{\mathrm{sp}}d\right)}{\sqrt{d}}\exp \left( i\pi /4\right){r}_2 d\theta . $$

where *φ* denotes the angle between the radial direction and the SPP propagating path and *d* is the distance from the secondary source to an arbitrary point *F*, as shown in Fig. 1b. Substituting Eq. (1) and Eq. (2) into Eq. (3) and Eq. (4), the SPP field distributions can be obtained and are given in Fig. 2a–d. The white dashed semicircle represents the semicircular slit, and the horizontal dashed line is drawn to clearly show the transverse shift of SPPs focus. It can be seen that the direction of the transverse shift of the SPP focus is always opposite for the left and right semicircular slits. For the spin-independent plasmonic lens, the SPP distribution is the superposition of the SPP fields generated by two semicircular slits, which can be written as ${E}_{\mathrm{sp}}\left(\rho, \theta \right)={E}_{\mathrm{sp}}^{\mathrm{L}}\left(\rho, \theta \right)+{E}_{\mathrm{sp}}^{\mathrm{R}}\left(\rho, \theta \right)$. Thus, the intensity of SPPs in the center is

5 $$ {\displaystyle \begin{array}{c}{I}_{s\mathrm{p}}\left(0,\theta \right)={\left|{E}_{\mathrm{sp}}\left(0,\theta \right)\right|}^2={\left|{E}_{\mathrm{sp}}^{\mathrm{L}}\left(0,\theta \right)+{E}_{\mathrm{sp}}^{\mathrm{R}}\Big(0,\theta \Big)\right|}^2\\ {}={I}_{\mathrm{sp}}^{\mathrm{L}}\left(0,\theta \right)+{I}_{\mathrm{sp}}^{\mathrm{R}}\left(0,\theta \right)+2\sqrt{I_{\mathrm{sp}}^{\mathrm{L}}\left(0,\theta \right){I}_{\mathrm{sp}}^{\mathrm{R}}\left(0,\theta \right)}\cos {\Delta \Phi}_{\mathrm{sp}},\end{array}} $$

where the phase difference is ΔΦ_sp_ = *k*_sp_(*r*_1_ − *r*_2_) − *π* and the term *π* results from the difference between the left and right spiral phases. To realize spin-independent focusing, the SPPs should interfere constructively in the center. Thus, the radiuses of the slits should satisfy

6 $$ \Delta r=\left(2n+1\right)\frac{\lambda_{\mathrm{sp}}}{2},\left(n=\cdots -2,-1,0,1,2,\cdots \right). $$

As presented in Fig. 2e and f, the SPP fields generated by LCP and RCP light are all focused in the center. The wavelength of incident light is 632.8 nm, and the corresponding wavelength of the SPPs *λ*_sp_ is 606 nm for the Au/air interface [12, 36]. The radiuses of the left and right semicircular slits are 5 μm and 4.697 μm. The normalized transversal and longitudinal distributions of the SPP focuses are extracted and compared in Fig. 2g and h. The spin-dependent transversal shifts of the SPP focuses in Fig. 2a–d disappear. The positions as well as the profiles of the SPP focuses generated by LCP and RCP light are exactly the same, which verifies the feasibility of the spin-independent plasmonic lens.

**Fig. 3:**
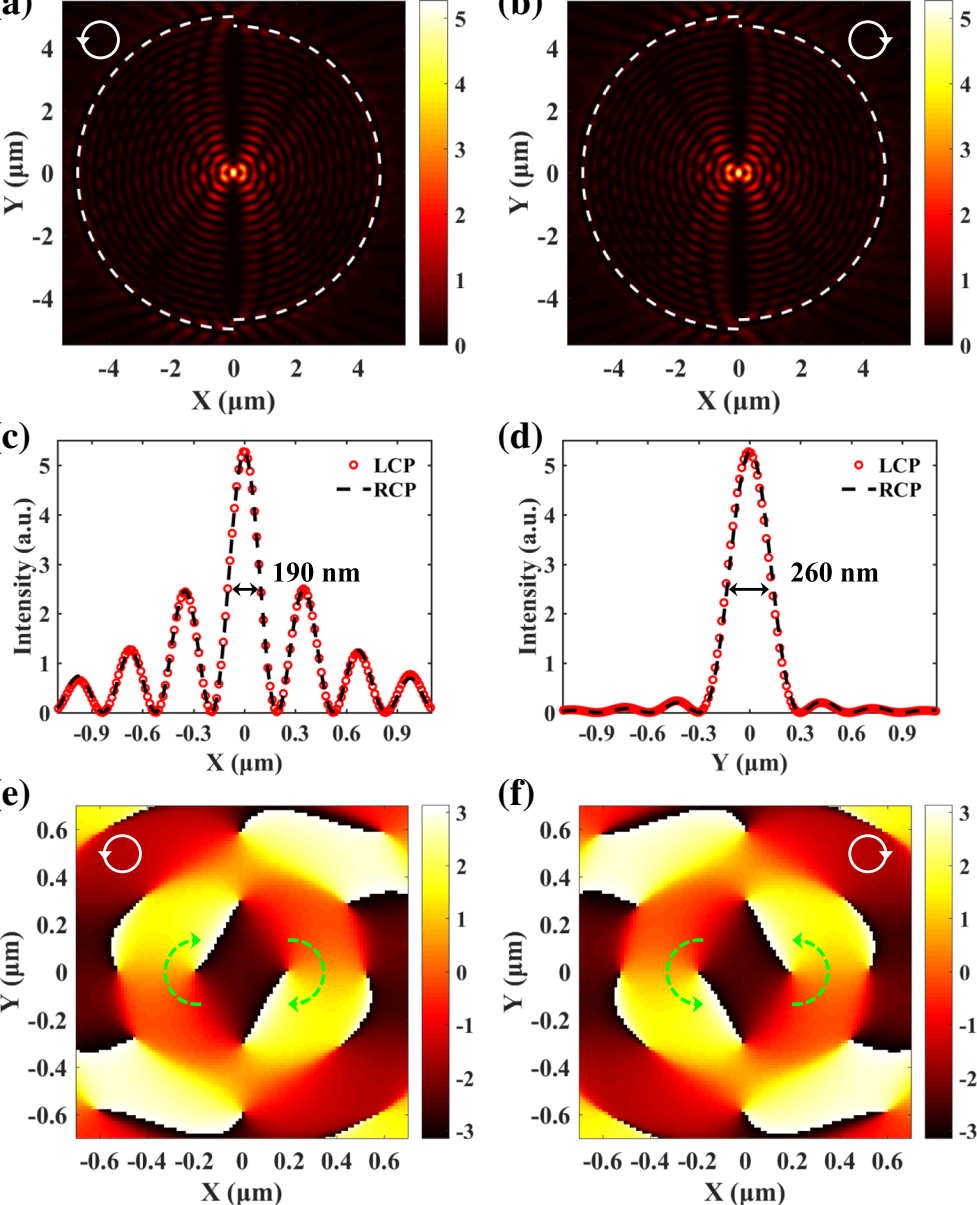
Simulated SPP field generated by LCP (**a**) and RCP (**b**) light. **c**, **d** The corresponding transversal and longitudinal distributions. The positions and profiles of the SPP focuses generated by LCP and RCP light are exactly the same. **e**, **f** The corresponding phase distributions around the focus. The two spiral phases with opposite directions in **e** and **f** can cancel out each other, which is the origin of spin-independent SPP focusing

Full-wave numerical simulations are also performed based on the finite-different time-domain (FDTD) method. The parameters are kept to be the same as the ones used in the analytical calculation with the Huygens-Fresnel principle. The simulated SPP distributions in Fig. 3a and b agree very well with the analytical results. The transversal and longitudinal distributions in Fig. 3c and d show that the full widths at half maximum (FWHM) of the focuses along the *x*- and *y*-direction (190 nm and 260 nm) are all smaller than half a wavelength. The position, the FWHM, and the intensity of the SPP focuses are all independent from the spin states of the incident light. The SPPs excited by the semicircular slits will gradually attenuate during propagation. The propagation loss is caused by the absorption in the metal [11, 12] and has been taken into consideration in the simulations by using a complex permittivity (*ε*_Au_ =  − 11.821 + 1.426*i*). Thus, the propagation loss does not affect the spin-dependent focusing of the SPPs. Figure 3 e and f give the phase distributions around the focal spot. As indicated by the green dotted arrows, two spiral phases with clockwise and counterclockwise directions counterbalance each other, which lead to the spin-independent SPP focusing. The flat phase in the center corresponds to the focusing area. It should be noted that the phase distributions of SPPs in Fig. 3e and f are different under different spin states of the excitation light. But they are central symmetry, which requires that the intensity distributions of SPPs should be center symmetry as well. To satisfy the center symmetry requirement, the SPP focuses generated by LCP and RCP light should both be located in the center. Thus, the spin-independent intensity distributions do not necessarily mean the phase distributions are spin-independent. Here, we mainly refer to the field intensity when saying spin-independent.

**Fig. 4:**
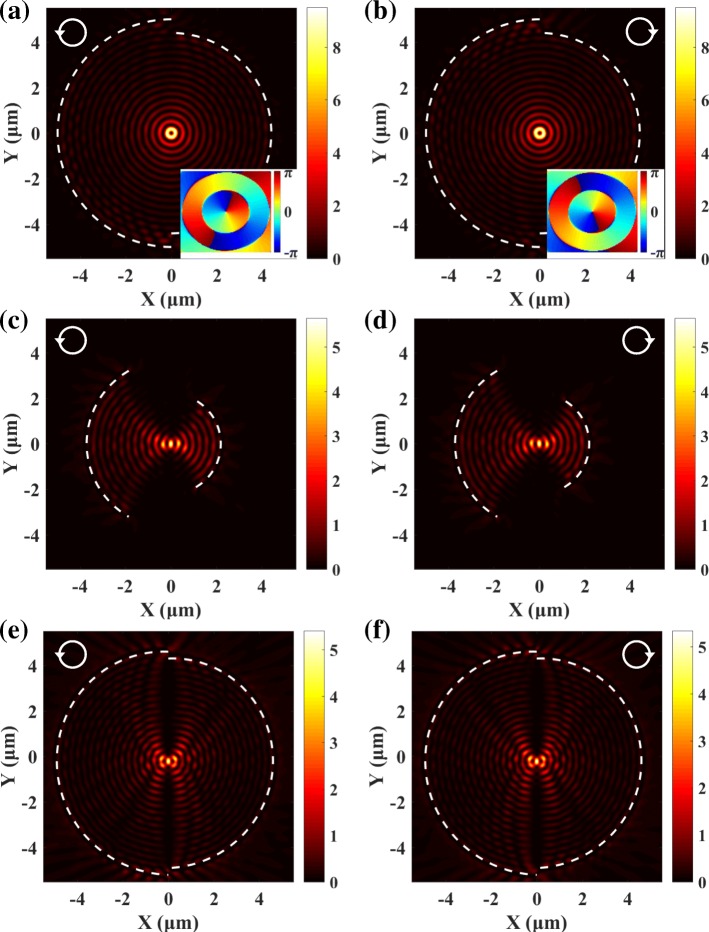
For semicircular slits with Δ*r* = *λ*_sp_, SPP vortexes excited by LCP (**a**) and RCP (**b**) exhibit opposite topological charges. The change of the radius and central angle will not affect the spin-independent SPPs focusing (**c**, **d**). The proposed approach is also suitable for spiral slits (**e**, **f**)

The evolutions of the SPP distribution with the difference of radiuses Δ*r* are revealed. When the radiuses satisfy Δ*r* = *nλ*_sp_, the two semicircular slits are equivalent to one circular slit with spiral phase varying from 0 to 2*π*. Taking Δ*r* = *λ*_sp_ as an example, the spin-dependent SPP vortexes can be obtained, as presented in Fig. 4a and b. The phase distributions in the insets of Fig. 4a and b show that the topological charge of SPP vortexes is *l* = 1 and *l* = − 1 for LCP and RCP light, respectively. Thus, the separation Δ*r* between the two semicircular slits has a great influence on the performance of the plasmonic lens. The two spiral phases can cancel each other out, and spin-independent SPP focusing can be accomplished only when Eq. (6) is satisfied. Moreover, according to Eq. (6), the radius and the central angle of the slits could not affect the focusing property of the plasmonic lens. For arc slits with a central angle 2*π*/3, *r*_1_ =3.7 μm and *r*_2_ =2.2 μm, $\Delta r=\frac{5}{2}{\lambda}_{\mathrm{sp}}$, and the SPPs excited by LCP and RCP light are all focused in the center, as shown in Fig. 4c and d. Furthermore, the proposed approach can be applied to the spiral slits. For a spiral slit described by ${r}_1\left(\theta \right)={r}_0+\frac{\theta }{\pi }{\lambda}_{\mathrm{sp}}$, adding another spiral slit with *r*_2_ = *r*_1_ − *λ*_sp_/2 can counterbalance the spiral phase and realize spin-independent SPP focusing. The SPP distributions in Fig. 4e and f demonstrate the versatility and robustness of the proposed approach.

### Spin-Independent SPP Focusing Based on Pancharatnam-Berry Phase

**Fig. 5:**
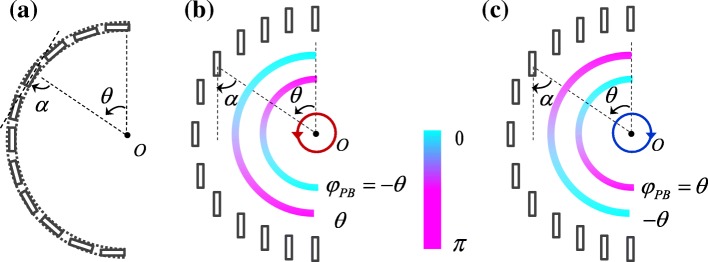
A semicircular slit can be divided into subwavelength rectangle slits (**a**). When the slits are vertically arranged, the PB phase generated by the each slit can be utilized to locally cancel the spiral phases generated by the LCP (**b**) and RCP light (**c**)

In the above discussions, we have treated the semicircular slit as a whole. As shown in Fig. 5a, a semicircular slit can be divided into subwavelength rectangle slits. In this way, the geometry Pancharatnam-Berry (PB) phase determined by the orientation angle of the slit is brought in [37, 38], which can be expressed as *φ*_PB_ = *σ*_m_*α*. Thus, the phase of SPPs generated by each subwavelength slit is:

7 $$ {\Phi}_{\mathrm{sp}}\left(\theta \right)={\sigma}_{\pm}\theta +{\varphi}_{\mathrm{PB}}. $$

The spiral phase can be canceled out locally by steering the PB phase distribution. In Fig. 5a, the PB phase is a constant *φ*_PB_ = *π*/2 and has no effect on the spiral phase. When the PB phase satisfies *φ*_PB_ = *σ*_m_*θ*, the spiral phase is counterbalanced locally and the phase of SPPs generated by each slit is Φ_sp_(*θ*) = 0. Thus, the subwavelength slits should be aligned along the vertical direction, as shown in Fig. 5b and c.

**Fig. 6:**
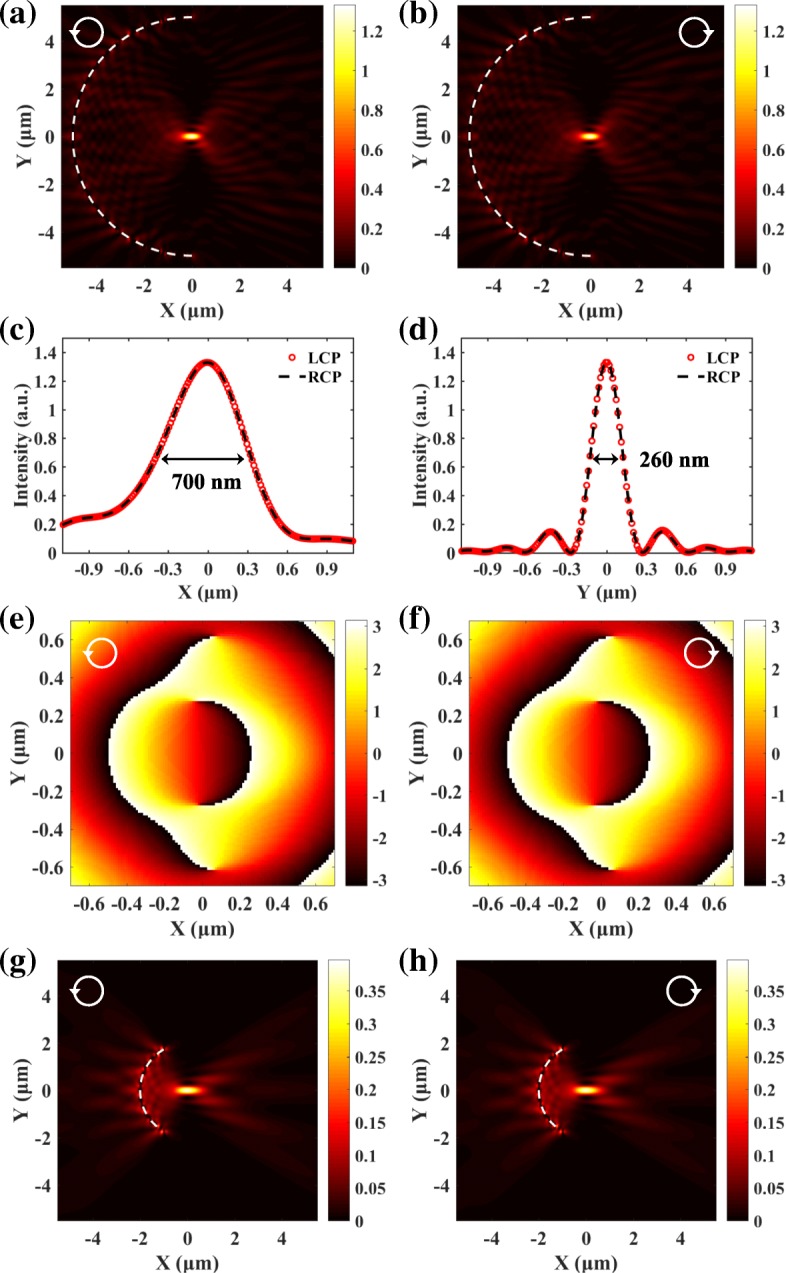
**a**, **b** The spin-independent SPP focusing for the lens consisted of subwavelength slits. **c**, **d** The transversal and longitudinal profiles of the SPP focus. **e**, **f** The corresponding phase distributions. **g**, **h** The spin-independent SPP focusing cannot be influenced by the change of the radius and central angle

The intensity distributions of SPPs generated by the spin-independent plasmonic lens consisted of vertical subwavelength slits are given in Fig. 6a and b. The width and length of the slits are 50 nm and 200 nm, respectively. The longitudinal and transversal profiles of the SPP focuses in Fig. 6c and d show that the position, the FWHM, and the intensity of the SPP focuses generated by the LCP and RCP light are indistinguishable. Compared with the SPP distributions in Fig. 3c and d, the transversal FWHM of the focus is about the same, while the longitudinal FWHM is more than three times larger. That is because SPPs generated by the opposite semicircular slit in Fig. 3c and d can effectively compress the transverse size of the SPP focus. Figure 6 e and f present uniform angular phase distributions around the focus, and no spiral phase is observed. That is because the spiral phase has been locally canceled by the PB phase. This is clearly different from the double semicircular slits approach which still preserves the spiral phases in Fig. 3e and f. The change of radius and central angle will not affect the focusing property of the SPP lens. Figure 6 g and h show the spin-independent SPP distributions generated by slits with a central angle 2*π*/3 and radius *r* = 2 μm.

## Conclusions

In conclusion, counterbalancing the spin-dependent spiral phase by introducing another spiral phase or Pancharatnam-Berry phase is the fundamental principle of spin-independent SPP focusing. The positions and profiles of SPP focuses generated by LCP and RCP light are exactly the same with the spin-independent plasmonic lens. This study further reveals that the spiral phase is a decisive factor in determining the focusing property of the semicircular plasmonic lens. Moreover, the proposed methods can be utilized to design polarization-independent devices in other frequency bands [39, 40] by scaling the structure.

## Methods

3D numerical simulations are performed with the commercial software Lumerical FDTD Solutions. In the simulation, semicircular slits with a width of 240 nm are etched on the 150-nm-thick gold film and the substrate is SiO_2_ with a refractive index of 1.46. The refractive index of the gold film can be obtained from the Johnson and Christy model [36]. The mesh accuracy is set as 3, and the corresponding size of each mesh cell is about 13 × 13 × 40 nm, which can achieve a good trade-off between accuracy, memory requirements, and simulation time. Perfectly matched layers (PML) with eight numbers of layers in the *x*-, *y*-, and *z*-directions are utilized as the boundary conditions to absorb the propagating SPP fields. Horizontally polarized light and vertically polarized light with a phase different *σ*_±_*π*/2 are utilized to synthesize the LCP and RCP light sources. And the light source illuminates the sample from the backside to avoid its influence on the excited SPPs. To obtain the profiles of SPP focus, a 2D field monitor is placed 50 nm above the gold film, which is within the decay length of SPPs.

## Abbreviations

FDTD: Finite-different time-domain
FT: Fourier transform
FWHM: Full widths at half maximum
LCP light: Left circularly polarized light
PB phase: Pancharatnam-Berry phase
RCP light: Right circularly polarized light
SHE: Spin Hall effect
SPPs: Surface plasmon polaritons

## References

[1] Pendry JB (2010). Negative refraction makes a perfect lens. Phys Rev Lett.

[2] Hell SW (2007). Far field optical nanoscopy. Science.

[3] Liu Z, Lee H, Xiong Y, Sun C, Zhang X (2007). Far-field optical hyperlens magnifying sub-diffraction-limited objects. Science.

[4] Kou SS, Yuan G, Wang Q, Du L, Balaur E, Zhang D, Tang D, Abbey B, Yuan X-C, Lin J (2016). On-chip photonic Fourier transform with surface plasmon polaritons. Light - Sci App.

[5] Hu D, Wang X, Feng S, Ye J, Sun W, Kan Q, Klar PJ, Zhang Y (2013). Ultrathin terahertz planar elements. Adv Opt Mater.

[6] Chen X, Huang L, Muhlenbernd H, Li G, Bai B, Tan Q, Jin G, Qiu CW, Zhang S, Zentgraf T (2012). Dual-polarity plasmonic metalens for visible light. Nat Commun.

[7] Khorasaninejad M, Chen WT, Devlin RC, Oh J, Zhu AY, Capasso F (2016). Metalenses at visible wavelengths: diffraction-limited focusing and subwavelength resolution imaging. Science.

[8] Khorasaninejad M, Capasso F (2017). Metalenses: versatile multifunctional photonic components. Science.

[9] Yu NF, Capasso F (2014). Flat optics with designer metasurfaces. Nat Mater.

[10] Sorger VJ, Oulton RF, Ma R-M, Zhang X (2012). Toward integrated plasmonic circuits. MRS Bull.

[11] Barnes WL, Dereux A, Ebbesen TW (2003). Surface plasmon subwavelength optics. Nature.

[12] Maier SA Plasmonics (2007). Fundamentals and applications.

[13] Ditlbacher H, Krenn JR, Schider G, Leitner A, Aussenegg FR (2002). Two-dimensional optics with surface plasmon polaritons. Appl Phys Lett.

[14] He X, Liu F, Lin F, Xiao G, Shi W (2019). Tunable MoS2 modified hybrid surface plasmon waveguides. Nanotechnology.

[15] Shi C, He X, Peng J, Xiao G, Liu F, Lin F, Zhang H (2019). Tunable terahertz hybrid graphene-metal patterns metamaterials. Opt Laser Technol.

[16] He X, Liu F, Lin F, Shi W (2018). Graphene patterns supported terahertz tunable plasmon induced transparency. Opt Express.

[17] He XY, Xia GN, Li F, Lin FT, Shi WZ (2019). Flexible properties of THz graphene bowtie metamaterials structures. Opt Mater Express.

[18] Schuller JA, Barnard ES, Cai W, Jun YC, White JS, Brongersma ML (2010). Plasmonics for extreme light concentration and manipulation. Nat Mater.

[19] Yin L, Vlasko-Vlasov VK, Pearson J, Hiller JM, Hua J, Welp U, Brown DE, Kimball CW (2005). Subwavelength focusing and guiding of surface plasmons. Nano Lett.

[20] Lerman GM, Yanai A, Levy U (2009). Demonstration of nanofocusing by the use of plasmonic lens illuminated with radially polarized light. Nano Lett.

[21] Li X, Zhang R, Zhang Y, Ma L, He C, Ren X, Liu C, Cheng C (2018). Slit width oriented polarized wavefields transition involving plasmonic and photonic modes. New J Phys.

[22] Bliokh KY, Gorodetski Y, Kleiner V, Hasman E (2008). Coriolis effect in optics: unified geometric phase and spin-hall effect. Phys Rev Lett.

[23] Gorodetski Y, Niv A, Kleiner V, Hasman E (2008). Observation of the spin-based plasmonic effect in nanoscale structures. Phys Rev Lett.

[24] Shitrit N, Nechayev S, Kleiner V, Hasman E (2012). Spin-dependent plasmonics based on interfering topological defects. Nano Lett.

[25] Wang S, Wang XK, Zhao F, Qu SL, Zhang Y (2015). Observation and explanation of polarization-controlled focusing of terahertz surface plasmon polaritons. Phys Rev A.

[26] Lee S, Kim K, Kim S, Park H, Kim K, Lee B (2015). Plasmonic meta-slit: shaping and controlling near-field focus. Optica.

[27] Zhang X, Xu Y, Yue W, Tian Z, Gu J, Li Y, Singh R, Zhang S, Han J, Zhang W (2015). Anomalous surface wave launching by handedness phase control. Adv Mater.

[28] Bao Y, Zu S, Liu W, Zhou L, Zhu X, Fang Z (2017). Revealing the spin optics in conic-shaped metasurfaces. Phys Rev B.

[29] Lin J, Mueller JP, Wang Q, Yuan G, Antoniou N, Yuan XC, Capasso F (2013). Polarization-controlled tunable directional coupling of surface plasmon polaritons. Science.

[30] Kim H, Park J, Cho SW, Lee SY, Kang M, Lee B (2010). Synthesis and dynamic switching of surface plasmon vortices with plasmonic vortex lens. Nano Lett.

[31] Xiao S, Zhong F, Liu H, Zhu S, Li J (2015). Flexible coherent control of plasmonic spin-hall effect. Nat Commun.

[32] Wang S, Wang S, Zhang Y (2018). Polarization-based dynamic manipulation of Bessel-like surface plasmon polaritons beam. Opt Express.

[33] Yin X, Chen L, Li X (2018). Polarization-controlled generation of Airy plasmons. Opt Express.

[34] Teperik TV, Archambault A, Marquier F, Greffet JJ (2009). Huygens-Fresnel principle for surface plasmons. Opt Express.

[35] Li X, Gao Y, Jiang S, Ma L, Liu C, Cheng C (2015). Experimental solution for scattered imaging of the interference of plasmonic and photonic mode waves launched by metal nano-slits. Opt Express.

[36] Johnson PB, Christy RW (1972). Optical constants of the noble metals. Phys Rev B.

[37] Genevet P, Wintz D, Ambrosio A, She A, Blanchard R, Capasso F (2015). Controlled steering of Cherenkov surface plasmon wakes with a one-dimensional metamaterial. Nat Nanotechnol.

[38] Wang S, Wang X, Zhang Y (2017). Simultaneous Airy beam generation for both surface plasmon polaritons and transmitted wave based on metasurface. Opt Express.

[39] Yuan Y, Zhang K, Ding X, Ratni B, Burokur SN, Wu Q (2018). Complementary transmissive ultra-thin meta-deflectors for broadband polarization-independent refractions in the microwave region. Photonics Res.

[40] Zhang K, Yuan Y, Zhang D, Ding X, Ratni B, Burokur SN, Lu M, Tang K, Wu Q (2018). Phase-engineered metalenses to generate converging and non-diffractive vortex beam carrying orbital angular momentum in microwave region. Opt Express.

